# The Comparison of Sensitivity and Specificity of ELISA-based Microneutralization Test with Hemagglutination Inhibition Test to Evaluate Neutralizing Antibody against Influenza Virus (H1N1)

**Published:** 2017-12

**Authors:** Ahmad TAVAKOLI, Farhad REZAEI, Gazal Sadat FATEMI NASAB, Fatemeh ADJAMINEZHAD-FARD, Zahra NOROOZBABAEI, Talat MOKHTARI-AZAD

**Affiliations:** Dept. of Virology, School of Public Health, Tehran University of Medical Sciences, Tehran, Iran

**Keywords:** Microneutralization assay, MicroNT-ELISA, Hemagglutination inhibition assay, Influenza virus, Serological assays

## Abstract

**Background:**

The most common serological assay to measure anti-influenza antibodies is hemagglutination inhibition (HI) assay. Recently, neutralizing antibodies against influenza virus infection or vaccination can also be detected using microneutralization assays and occasionally, have greater sensitivity than the standard HI assays. The study aimed to compare the sensitivity and specificity of ELISA-based microneutralization (microNT-ELISA) and conventional HI assays in order to detect influenza H1N1 virus antibodies.

**Methods:**

MicroNT-ELISA was set up according to the WHO Manual on Influenza Diagnosis and Surveillance in Virology Department of Tehran University of Medical Sciences for the detection of neutralizing antibodies against H1N1 influenza virus in 2013. Fifty serum samples were analyzed with both HI and microNT-ELISA assays. Correlation between methods was calculated by linear regression analysis.

**Results:**

The linear correlation coefficient squares, R2, of microNT-ELISA and HI test was 0.61 (*P*<0.0001) and we observed a high index of coincidence between the two tests. According to McNemar’s test, there was no statistically significant difference between these two assays (*P*>0.05).

**Conclusion:**

The sensitivity and specificity of microNT-ELISA assay were high (87% and 73%, respectively) and closely related to gold standard test results. Therefore, microNT-ELISA is recommended as an alternative or complementary test to conventional HI assay for serological and epidemiological purposes.

## Introduction

Influenza virus infection remains a major public health threat, which causes significant human morbidity and mortality during seasonal epidemics and pandemics. There are several techniques for detection of influenza virus infection. Serological assays are important tools in this way. These techniques are used to surveillance, developing and evaluation of vaccine, seroepidemiological studies, and sometimes in diagnosis, especially in the reemergence of new influenza virus strains (1).

After exposure to influenza either via infection or vaccination, the humoral immune response will start to produce specific antibodies against particular viral antigens. These antibodies can be measured by different serological methods about 2–3 wk after the onset of symptoms (2). Hence, serological approaches able to confirm the past infection without virologically confirmed symptomatic influenza. Demonstration of a significant increase in antibody titer (≥4-fold) between acute- and convalescent-phase sera leads to diagnose of a recent influenza infection, even when attempts to virus detection are unsuccessful (3). In addition to the retrospective diagnostic value, serological assays such as neutralization and HI have important roles in epidemiological and immunological surveys, as well as in assessment of vaccine immunogenicity (4).

HI is the most common used assay for quantifying anti-influenza antibodies. Capability of influenza viruses to agglutinate chicken RBC was first proposed by Hirst (5). HI is commonly considered as the gold standard in influenza virus serology (6) and is used for diagnosis of influenza virus infections (7–9), to determine vaccine immunogenicity (10, 11) and for seasonal surveillance (12). However, the HI titer can be influenced by the distinguished expression of sialic acid receptors on the membranes of different red blood cells, which may affect the binding affinity. The result of HI test is also affected by the type of RBC (13, 14). Furthermore, removing of nonspecific inhibitors in sera specimens is an inevitable step for HI test (15). Identification of neutralizing antibodies without the ability to inhibit hemagglutination can be also considered one of limitations of HI assay (16). On the other hand, HI assay is less sensitive for detection of antibodies against avian influenza viruses, especially H5N1 and H3N2 subtypes (17). To overcome the above limitations by HI assay, microneutralization assays have developed due to they can detect functional neutralizing antibodies to influenza virus infection or vaccination and occasionally, have shown greater sensitivity than the HI assays (17, 18).

The microNT-ELISA assay is based on the conventional serum neutralization test, but ELISA performs the measurement for the detection of virus-infected cells. This method can divide into three steps: determination of the tissue culture infectious dose (TCID), virus microneutralization assay, and ELISA. Using the microNT-ELISA assay, the results are achieved within 2 d (19, 20).

In this study, microNT-ELISA was set up according to the WHO Manual on Influenza Diagnosis and Surveillance (19) in Virology Department of Tehran University of Medical Sciences for the detection of neutralizing antibodies against H1N1 influenza virus. In addition, sensitivity and specificity of microNT-ELISA assay were compared with HI assay.

## Materials and Methods

Between Nov to Dec 2013, 50 serum samples were collected from staff members of the virology department of Tehran University of medical sciences. Since our aim was to measure antibodies levels by two mentioned assays, individuals with different levels of antibodies were required. Patients with acute infection may have no detectable antibodies level during sample collection. However, staff members of the virology department enrolled in this study may have desired antibodies in different levels due to history of influenza vaccination, as well as being at higher risk of exposure to influenza virus.

The present study followed the principles of the Declaration of Helsinki and was approved by the local Ethics Committee at Tehran University of Medical Sciences, Tehran, Iran. The participants were informed about the current study, and informed consent was obtained from all of the persons prior to their enrollment.

### HI assay

The method of the HI assay was performed according to the WHO protocol (19). Fifty serum samples were tested by this procedure. For removing non-specific hemagglutination inhibitors, sera samples firstly were treated with receptor-destroying enzyme (RDE), incubated for overnight in a 37 °C water bath, and heated in a water bath at 56 °C for 30 min to inactivate RDE. 96-well microtiter plates performed the test. Positive control serum was prepared from sera of patients whom H1N1 infection was previously confirmed by polymerase chain reaction (PCR), cell culture and sequencing. In fact, sera specimens obtained 1–2 months after their infection; then, the exact antibody titer against H1N1 influenza virus determined using reference virus strain and the gifted antibodies by WHO. In addition, negative control serum was obtained from patients with respiratory tract infection who were negative in both PCR and cell culture for influenza.

In summary, 25 μl of phosphate-buffered saline (PBS) was added from rows B to H prior to addition of 50 μl of RDE-treated sera from rows A to H. Serial twofold dilutions were made by transferring 25 μl amounts from the first row to next rows, and in the final row 25 μl was discarded. Antigen-containing four hemagglutinations (HA) units/25 μl of the reference virus strain, A/California/2009 H1N1 pdm09-like virus, was then added to each well and the plates were incubated at room temperature for 60 min. Afterwards, 50 of guinea pig red blood cells (0.5%) were added to each well. The plates were incubated again at room temperature for 1 hour. The HI titer was presented as the highest reciprocal serum dilution that entirely inhibited the hemagglutination of 4 HA units of the virus. HI, titers of 1:40 and higher were considered to be positive (21).

### ELISA-based microneutralization assay

The method of the microNT-ELISA test was carried out by a procedure described previously (17) and WHO protocol (19). For microNTELISA test, all 50-serum samples that were already treated with RDE were also heat inactivated at 56 °C for 30 min. The sera were twofold serially diluted in duplicate in 96-well microtiter plates, and incubated with 50 μl virus suspension (100 TCID50/ml in diluent). The TCID50 of stock virus was calculated by the method (22). The reference virus strains A/California/2009 H1N1 pdm09-like virus was used for performing micro-NT ELISA, similar to HI assay. The plates were incubated at 37 °C for 2 h and 5% CO2. Afterwards 100 μl of Madin-Darby canine kidney (MDCK) cells (1.5 × 10^5^/ml) were added to each well. The plates included four wells containing 50 μl of diluted virus, 50-μl diluent and 100 μl of MDCK cells as positive controls and 4 wells containing 100 diluent and 100 μl of MDCK cells as cell control. After overnight incubation (18–20 h) at 37 °C and 5% CO2, the medium was removed from the wells and the monolayers were fixed with cold fixative (acetone 80% in PBS 1:5) for 10 min. In order to detect virus-infected cells, an ELISA was performed to determine the titer. After three times washing, the fixed plates with the wash buffer (PBS, 0.1% Tween 20), the anti-influenza A NP mouse monoclonal antibody was used at a 1: 4000 dilution in blocking buffer (PBS, 1% bovine serum albumin and 0.1% Tween 20). Then, 100 μl volumes per well were added. Following a one hour incubation period at room temperature, the plates were washed four times with PBS (pH = 7.4). About 100 μl of horseradish peroxidase-conjugated goat anti-mouse IgG (diluted 1:2000 in blocking buffer) was then added to all wells. The plates were allowed to incubate 1 h at room temperature. After that, the solution was removed and the wells were washed five times with wash buffer (PBS) and then filled with 100 μL of freshly prepared substrate solution (10 mg of o-phenylenediamine dihydrochloride per 20 ml of 0.05 M phosphate-citrate buffer, pH 5.0). The reaction was stopped after 5–10 min by the addition of 100 μl stop solution (0.5 N sulfuric acid) per well. The absorbance was measured by absorbance microplate reader BioTek at 490 (A490) nm.

The virus neutralization endpoint titer of each serum was calculated using the following equation:

X=[(average A490 of virus control wells)−(average A490 of cell control wells)]/2+(average A490 of CC wells).

All the values less than this reference are considered positive for neutralization activity. All sera were tested twice on separate days and the final titer was the average value of two separate runs.

## Results

**Fig. 1:**
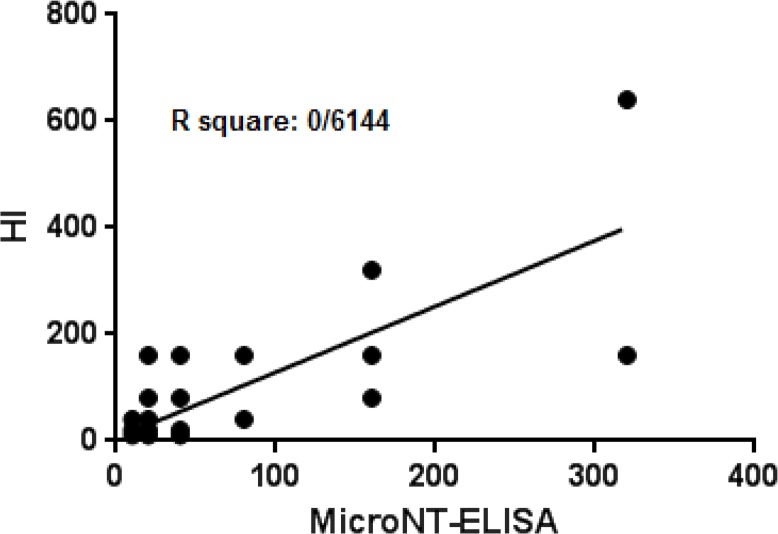
Correlation Between HI and microNT-ELISA assays (p<0/0001, *r*^2^=0/61)

Serum samples of 50 subjects (age, 23 to 62 yr; mean age, 34/7 yr, 62% male and 38% female) were analyzed. Statistical analysis was performed using GraphPad Prism version 6.01 and SPSS version 22 software. According to formula (sensitivity=number of true positives/number of true positives + number of false negative, specificity=number of true negatives/number of true negatives + number of false positives), sensitivity and specificity of microNT-ELISA were calculated 87% and 73%, respectively. Correlation between methods was calculated by linear regression analysis. The linear correlation coefficient squares, R2, of microNT-ELISA and HI test was 0.61 (*P*<0.0001). It was observed a high index of coincidence between the two tests (Fig. 1). McNemar test was not assessed the statistically significant difference between two assays (*P*>0.05). There was also no significant relationship between gender and microNT-ELISA and HI assays (Table 1). Antibody titers ≥1:40 considered as positive samples since it has defined as seroprotection.

**Table 1:** Comparison of positive and negative results of HI and microNT-ELISA

***Sex***	***HI (Count and Percent)***		***Total***	***MicroNT-ELISA (Count and Percent)***		***Total***
	**Positive**	**Negative**		**Positive**	**Negative**	
Male	8 (25.8)	23 (74.2)	31 (100)	14 (45.2)	17 (54.8)	31 (100)
Female	8 (42.1)	11 (57.9)	19 (100)	8 (42.1)	11 (57.9)	19 (100)
Total	16 (32)	34 (68)	50 (100)	22 (44)	28 (56)	50 (100)

## Discussion

Detection of strain-specific antibodies by the microneutralization assays is highly sensitive and specific (23). These assays consist of three separate steps: a virus–antibody reaction step (mixing the virus with different dilutions of serum sample), an inoculation step (inoculation of mixture from previous step into the appropriate host system), and a read-out step (a procedure for detection of virus or viral antigen). The absence of infectivity is regarded as a positive neutralization reaction and demonstrates the presence of virus-specific antibodies in the serum specimens (1).

The neutralization assay measures cytopathic effects (CPEs) caused by virus infection (24). Currently, microneutralization assays using MDCK cells in microtiter plates are the preferred high throughput methods for detecting neutralizing antibodies. Some studies were performed for evaluating the sensitivity and specificity of microneutralization assays for different aims. For instance, collected samples were studied before and after vaccination against H1NA influenza in HIV patient; HI and microneutralization assay performed for assessment of antibody levels. Estimated seroprotection of microneutralization assay was 70.2%, while HI assay assessed 45.2% (25). More commonly, ELISA has been included in microneutralization assays for detecting of viral antigen such as nucleoprotein expressed in infected cells. By comparison with the cytopathic effect (CPE)-based neutralization tests (Nt-*CPE*), ELISA-based neutralization assays tend to be less variable (26, 27).

The purpose of current study was to compare sensitivity and specificity of microNT-ELISA and conventional HI assays in order to detect of influenza H1N1 virus antibodies. Although the HI assay is considered the gold standard method for serologic diagnosis of human influenza infections, the assay has been reported to be less sensitive to detect antibody responses to influenza H3N2 and avian viruses in mammalian sera (17, 28, 29).

Our results have been shown a high correlation between HI and microNT-ELISA assays. Due to lack of statistical significance calculated by McNemar test between microNT-ELISA and HI assays, the results of microNT-ELISA assay are closely related to HI assay as a gold standard test. A direct comparison of an HI assay and the microNT-ELISA assay was performed, and the microNT-ELISA assay was substantially more sensitive in detecting human antibodies to H5N1 virus in infected persons (17). The quality of the MDCK cells is most important for maximizing the proper use of the microNT-ELISA. The influenza virus replication levels also are affected by the sublineages of MDCK cells. In addition, a low passage number was also eligible for appropriate replication of the test viruses and it can influence quality of virus replication so that less than 25 passage number is optimal to preparation of microNT-ELISA assay (17). MDCK cells at passage 13, used in our study.

Neutralizing antibodies and hemagglutination inhibition antibodies were measured following by seasonal influenza vaccination (30). Measured values for seroprotection using HI were found to show higher rates than that of measured by microNT-ELISA. Although the conventional HI test could detect higher antibody titers, HI-antibodies might not fully reflect neutralization properties or may occur because of antibody cross-reactivity among various virus strains.

Several studies have found antibodies that recognize different influenza viruses (31–33). HA stem region of H1, H2, H5, H6, H8, H9, H11, H12, H13, H16 influenza A virus is the binding site for some of these antibodies, while the other antibodies bind to most group H3, H4, H7, H10, H14, H15 influenza A viruses stem region (34). In contrast, most microNT-ELISA titers in prevaccination sera were below 1:10, indicating low cross-reactivity to different virus strains (30). Therefore, microNT-ELISA is recommended for detection of antibodies against influenza virus. HI is the most common serological test used to detect anti-influenza antibodies. Although it is ease of performance, technical errors may affect its accuracy and sensitivity of HI insensitivity and inaccuracy from technical errors and the ability of the species or the quality of the RBCs to affect the results are of concern. Microneutralization tests were developed to overcome the limitations of HI assay, and the assays were shown to be more sensitive than HI for detecting antibody titers against the influenza virus. Since antibodies that neutralize virus may not inhibit hemagglutination, they could not be detected in the HI assay. These include neutralizing antibodies targeting the HA stem region, neuraminidase, or the M2 ectodomain. On the other hand, some antibodies that inhibit hemagglutination may not have virus-neutralizing activity and therefore not detected by the microNT-ELISA assay. Furthermore, the virus strains and the host source of RBC can affect the binding avidity in the HI assay.

Recently isolated human influenza viruses A (H3N2) appear to have lost the ability to agglutinate chicken erythrocytes (35).

Finally, there are nonspecific hemagglutination inhibitors present in human sera, designated as α, β, and δ. These inhibitors should be removed during the HI assay but not in microNT-ELISA assay. Since β and δ inhibitors may also have neutralizing activity, this activity might be partly related to these factors.

Because of using live viruses in microNT-ELISA assay, there are some limitations when highly pathogen viruses such as H5N1 influenza virus are utilized. In this regard, BSL3 laboratory equipment is required. Eventually, we mentioned the profits of utilizing microNT-ELISA assay.

Transmissions of highly pathogenic H7N9 and H5N1 avian influenza viruses to human populations have triggered the seroepidemiological studies. Occasionally, detection of avian influenza virus antibody among human by HI assay failed even when virus isolated with virus cultured. HI, assay could not distinguish specific antibodies to different avian influenza viruses and showed high cross-reactivity. In contrast, microNT-ELISA assay showed lower cross-reactivity and is better in this respect. Moreover, HI assay only presents total neutralizing antibodies, but microNTELISA assay can distinguish specific antibodies into IgG or IgM separately.

## Conclusion

The sensitivity and specificity of microNTELISA assay are high (87 and 73%, respectively) and closely related to gold standard test results. Therefore, we recommend the microNT-ELISA test as an alternative or complementary test to conventional HI test in order to serological and epidemiological purposes and assessment of influenza vaccines immunogenicity.

## Ethical considerations

Ethical issues (Including plagiarism, informed consent, misconduct, data fabrication and/or falsification, double publication and/or submission, redundancy, etc.) have been completely observed by the authors.
